# Sensitivity-Enhanced SPR Sensor Based on Graphene and Subwavelength Silver Gratings

**DOI:** 10.3390/nano10112125

**Published:** 2020-10-26

**Authors:** Lu Kong, Jiangtao Lv, Qiongchan Gu, Yu Ying, Xiaoxiao Jiang, Guangyuan Si

**Affiliations:** 1 College of Control Engineering, Northeastern University at Qinhuangdao, Qinhuangdao 066004, China; kl13043130659@163.com; 2 College of Information Science and Engineering, Northeastern University, Shenyang 110004, China; 3 Hebei Key Laboratory of Micro-Nano Precision Optical Sensing and Measurement Technology, Qinhuangdao 066004, China; guqiongchan@163.com; 4 College of Information & Control Engineering, Shenyang Jianzhu University, Shenyang 110168, China; yingyu@sjzu.edu.cn

**Keywords:** surface plasmon resonance, graphene, subwavelength grating, sensitivity

## Abstract

A novel surface plasmon resonance (SPR) sensor with graphene and subwavelength gratings is proposed to improve the sensing performance. A series of numerical analyses were performed to investigate the effect of structural parameters on the sensing performance, such as minimum reflectance at resonance (MRR), full width at half maximum (FWHM), and resonance angle. The results indicated that near-zero MRR (2.9 × 10^−6^) and narrow FWHM (about 3.5 deg) could be obtained by optimizing the geometrical parameters. Moreover, the influence of the number of graphene layers on sensitivity was also studied. The maximum sensitivity of the designed sensor could reach 192 deg/refractive index unit (RIU), which is a great enhancement compared to the silver-only SPR sensor. In addition, ethylene glycol solutions with different refractive indices were detected. The results showed that the sensitivity of the sensor could reach 220.67 deg/RIU, and the proposed sensor had excellent linearity between the resonance angle and refractive index, enabling extensive potential practical sensing applications.

## 1. Introduction

Surface plasmon resonance (SPR), an optical phenomenon that is considerably sensitive to small changes in the analyte refractive index, has occupied a critical position among optical technologies [1,2]. As has been extensively and intensively documented in the literature, SPR sensors have been diffusely applied in different detection applications, including environmental monitoring [3], drug discovery [4], food allergen detection [5], and gas sensing [6], because of advantages such as label-free detection, low cost, real-time measurement, high sensitivity, and so on [7]. To excite SPR at a metal–dielectric interface, many configurations have been proposed, including prism coupling [8,9], waveguide coupling [10,11,12,13,14], fiber coupling [15], and grating coupling [16]. Over the past few decades, various strategies have been developed to increase the performance of SPR sensors, including bimetallic films [17,18], metallic nanowires [19], nanohole arrays [20], metal nanorods [21], nanoparticles [22], nanogratings [23,24,25], and so on. 

Graphene, a promising two-dimensional (2D) material with excellent electrical [26], mechanical [27], thermal [28], and optical [29] properties, has attracted great interest in various fields. Graphene has been integrated with SPR systems as a biomolecular recognition element (BRE) to functionalize metallic film to improve the sensing performance because it possesses rich π conjugation structure and high surface-to-volume ratio [30,31]. For the last few years, various designs consisting of graphene have been reported to enhance the performance of SPR sensors. Wu et al. [31] reported a highly sensitive SPR sensor based on graphene–gold structure, which achieved a sensitivity enhancement of 25% compared to the bare gold-based conventional SPR sensor. Choi et al. [32] proposed a SPR imaging biosensor based on graphene–silver (Ag) structure and found that the imaging sensitivity was amplified more than three times in detecting DNA hybridization with single-layered graphene on the Ag film. Verma et al. [33] presented a SPR biosensor based on graphene–silicon–gold structure, which achieved more than double the sensitivity enhancement with 40 nm gold, 7 nm silicon layers, and two layers of graphene. Moreover, SPR sensors based on rhodium–Ag–graphene structure [34], SnSe–graphene hybrid structure [35], and graphene tri sheets [36] have been proposed in recent years. In addition, SPR sensors based on graphene-decorated nanostructures have been developed, such as graphene–gold nanoparticles [37] and graphene grating structures [38,39,40]. 

**Figure 1:**
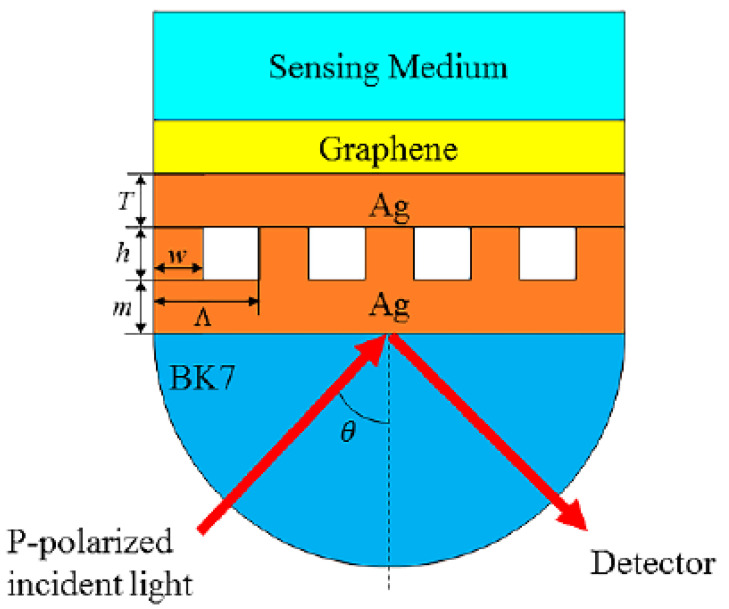
Schematic diagram of the proposed surface plasmon resonance (SPR) sensor with Ag gratings and graphene.

In this paper, a novel SPR sensor consisting of graphene and subwavelength silver gratings is proposed, as shown in Figure 1. The performance of the proposed SPR sensor was investigated through COMSOL Multiphysics commercial software based on the finite element method (FEM). The effects of metal film thickness and grating structural parameters, including grating height, grating width, and grating period, on the sensing performance, such as minimum reflectance at resonance (MRR), full width at half maximum (FWHM), and resonance angle, were studied to optimize the structural parameters. Under optimal conditions, the sensitivity of the designed sensor with different number of graphene layers was calculated. The simulation results indicated that the highest sensitivity could reach 192 deg/RIU, which is a significant increase compared to the Ag-only SPR sensor. In addition, ethylene glycol solutions with different concentrations were detected with a maximum sensitivity of 220.67 deg/RIU, which is an enhancement of 80.21%. The proposed sensor had excellent linearity between the resonance angle and refractive index in the range between 1.333 and 1.360.

## 2. Theoretical Model 

A schematic diagram of the proposed SPR sensor consisting of graphene and subwavelength grating structure is depicted in Figure 1. A lower thin Ag film (thickness presented by *m*) was placed on a BK7 prism. In addition, a subwavelength grating structure was placed on the lower silver film, with the structural parameters of grating height, grating period, and grating width indicated by *h*, *Λ*, and *w*, respectively. An upper thin metal film was coated on the grating structure, the thickness of which is shown by *T*. In this work, Ag was chosen as the material for the upper and lower metallic films and subwavelength grating structure. Generally, a thin Ag film with a sharp SPR curve will yield higher sensitivity and narrower reflectance [41]. The graphene layer covered by a sensing medium layer was placed on the upper Ag film. Under the angular interrogation mode, a *p*-polarized light with operation wavelength of 633 nm was incident on the BK7 prism. This structure was conducted via a two-dimensional model and investigated using COMSOL Multiphysics. Meanwhile, Floquet periodic conditions were arranged for the lateral boundaries, and physics-controlled meshes with extremely fine sizes were employed in the simulations in order to ensure the most accurate results. 

Here, BK7 glass was used as a coupling prism, and its refractive index can be determined from the following expression [42]:

(1) $$n_{\mathrm{BK}7}={\left(\frac{1.03961212\lambda^{2}}{\lambda^{2}-0.00600069867}+\frac{0.231792344\lambda^{2}}{\lambda^{2}-0.0200179144}+\frac{1.01046945\lambda^{2}}{\lambda^{2}-103.560653}+1\right)}^{1/2},$$

According to the Drude–Lorentz model, the complex refractive index of silver is obtained as follows [43]:

(2) $$n_{Ag}={\left(1-\frac{\lambda^{2}\lambda_{c}}{\lambda_{p}^{2}(\lambda_{c}+i\lambda )}\right)}^{1/2},$$

where $\lambda_{p}$ (=1.4541 × 10^−7^ m) and $\lambda_{c}$ (=1.7614 × 10^−5^ m) represent the plasma and collision wavelengths of Ag, respectively. The refractive index of graphene is calculated as follows [44,45]:

(3) $$n_{G}=3+\frac{iC}{3}\lambda ,$$

where the constant $C\approx 5.446\;\mu m^{-1}$ [46]. In all the above equations, λ represents the vacuum wavelength of the incident light. The graphene layer thickness is equal to L×0.34 nm, where *L* is the number of graphene layers. The refractive index of the sensing medium is given as $n_{s}=1.33+\Delta n$, where Δn denotes the refractive index shift in the sensing medium layer.

## 3. Results and Discussion

### 3.1. Performance Parameter Setup

In this study, the performance of the proposed sensor was measured by three considerably important aspects: MRR, FWHM, and sensitivity. MRR represents transformation of the intensity of incident light to surface plasmons (SPs), with a smaller MRR meaning stronger excitation of SP waves [47,48,49,50]. FWHM is a geometrical interpretation of the SPR curve width. Narrower FWHM will yield higher detection accuracy, which benefits precise measuring of resonance angle [48]. The sensitivity (*S*) of the SPR sensor is obtained as the ratio of the resonance angle shift ($\Delta \theta_{SPR}$) to the refractive index change (Δn) in the sensing medium layer [49]. For a given change (Δn), $\Delta \theta_{SPR}$ should be as large as possible:

(4) $$S=\frac{\Delta \theta_{SPR}}{\Delta n}.$$

In the next section, the influence of the metal film thickness and the grating structural parameters, including grating height, grating width, and grating period, on the resonance angle, MRR, and FWHM will be studied to optimize the design and obtain the best performance.

### 3.2. Evaluation of Metal Film Thickness on Sensor Performance

**Figure 2:**
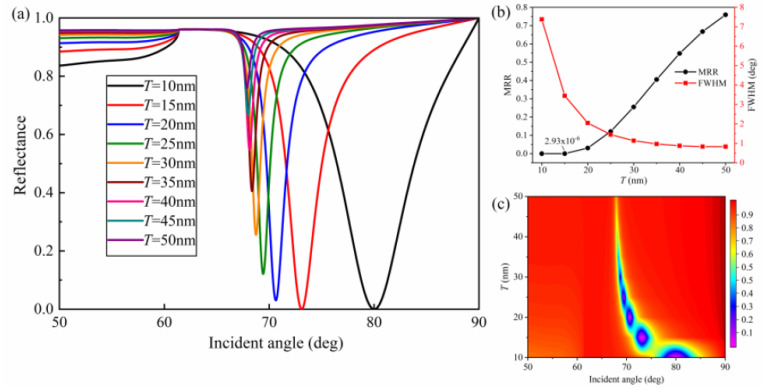
Influence of the upper silver film thickness (*T*) on reflectance: (**a**) SPR curves for various upper silver film thicknesses; (**b**) minimum reflectance at resonance (MRR) and full width at half maximum (FWHM) as a function of *T*; (**c**) contour plot of the reflectance (*m* = 15 nm, *w* = 50 nm, *h* = 50 nm, *Λ* = 100 nm, and *L* = 1).

The initial structural parameters of the proposed SPR sensor were as follows: *m* = 15 nm, *h* = 50 nm, *w* = 50 nm, *Λ* = 100 nm, *T* = 10 nm, and *L* = 1. As a high-performance SPR sensor should exhibit small reflectance at resonance, the grating geometrical parameters and film thickness were first optimized to achieve minimum MRR and a narrow FWHM. According to the SPR curves and the contour plot of reflectance in Figure 2a,c, the upper Ag film thickness (*T*) played a significant role in resonance angle, FWHM, and MRR. With the increase of *T* from 10 to 50 nm, the SPR curves shifted toward smaller resonance angle. In addition, Figure 2b quantitatively indicates the variation of FWHM and MRR with respect to different upper Ag film thickness (*T*) at *λ* = 633 nm. With the increase of the upper Ag film thickness, the SPR curves became narrower but the MRR got larger, which means the excitation of SPs was weak. Overall, the MRR became larger with increasing *T*, but the closest near-zero MRR equal to 2.93 × 10^−6^ was obtained when the upper silver film thickness was 15 nm with a narrow FWHM (≈3.45 deg).

**Figure 3:**
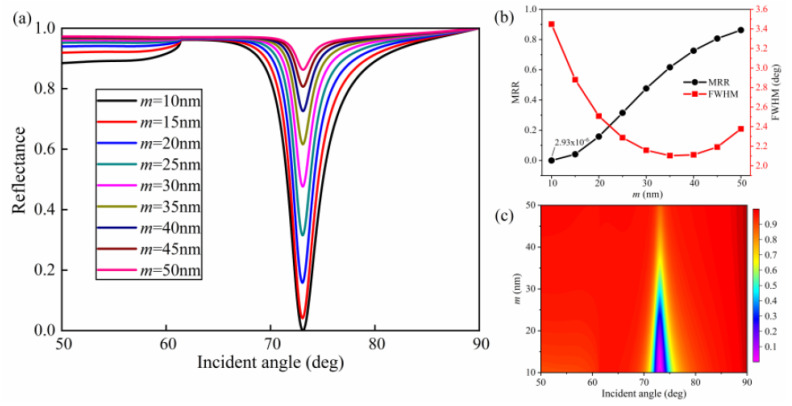
Influence of the lower silver film thickness (*m*) on reflectance: (**a**) SPR curves of various lower silver film thicknesses; (**b**) MRR and FWHM as a function of *m*; (**c**) contour plot of the reflectance (*T* = 15 nm, *w* = 50 nm, *h* = 50 nm, *Λ* = 100 nm, and *L* = 1).

To investigate the effect of the lower Ag film thickness (*m*) on the nature of SPR curves, Figure 3 shows the SPR curves, FWHM, and MRR as well the contour plot of reflectance with different values of *m*. As can be seen from Figure 3a,c, the resonance angle barely changed, and the value of resonance angle was about 73.10 deg with increasing *m*. However, FWHM and MRR of the SPR curves showed fluctuation as *m* changed. From Figure 3b, it is obvious that FWHM became narrower when the lower silver film thickness increased from 10 to 35 nm, which means higher detection accuracy. However, MRR continuously increased, meaning it would induce weaker coupling of SPs. The optimal value of *m* screened by ensuring near-zero reflectance and a narrow FWHM was 10 nm.

### 3.3. Evaluation of Grating Strutural Parameters on Sensor Performance

**Figure 4:**
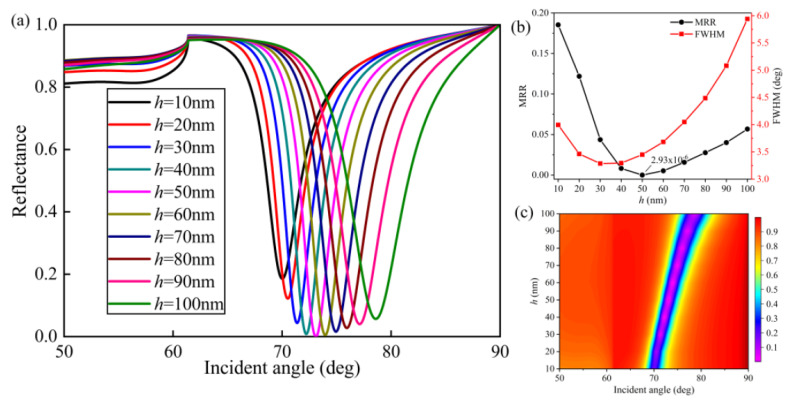
Influence of the grating height (*h*) on reflectance: (**a**) SPR curves of various grating heights; (**b**) MRR and FWHM as a function of *h*; (**c**) contour plot of the reflectance (*T* = 15 nm, *m* = 10 nm, *w* = 50 nm, *Λ* = 100 nm, and *L* = 1).

**Figure 5:**
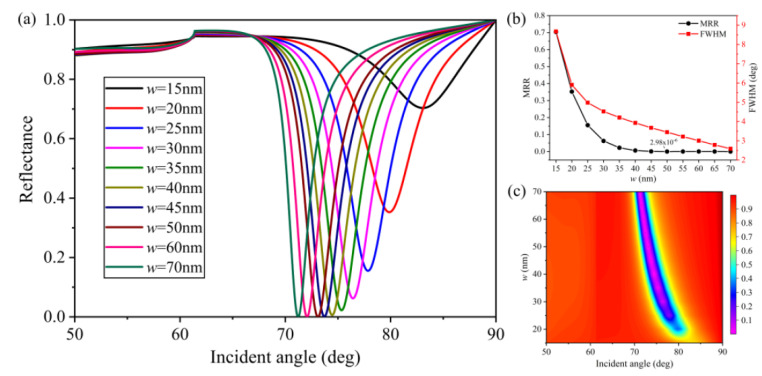
Influence of the grating width (*w*) on reflectance: (**a**) SPR curves of various grating widths; (**b**) MRR and FWHM as a function of *w*; (**c**) contour plot of the reflectance (*T* = 15 nm, *m* = 10 nm, *h* = 50 nm, *Λ* = 100 nm, and *L* = 1).

**Figure 6:**
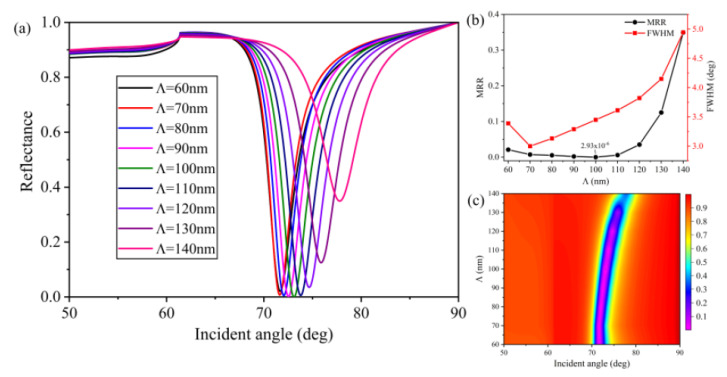
Influence of the grating period (*Λ*) on reflectance: (**a**) SPR curves of various grating periods; (**b**) MRR and FWHM as a function of *Λ*; (**c**) contour plot of the reflectance (*T* = 15 nm, *m* = 10 nm, *h* = 50 nm, *w* = 50 nm, and *L* = 1).

Figure 4, Figure 5 and Figure 6 illustrate the effect of grating structural parameters, including grating height (*h*), grating width (*w*), and grating period (*Λ*), on sensor performance. We increase *h* from 10 to 100 nm with a 10 nm step size, as presented in Figure 4, while the resonance angle redshifted from 70.02 to 78.59 deg. It can be seen from Figure 4b that the MRR decreased from 0.18 to 2.93 × 10^−6^ as *h* increased from 10 to 50 nm, revealing that the SPR phenomenon was getting more pronounced. The minimum FWHM was 3.287 deg at 30 nm, and the minimum MRR was at 50 nm. Although these two values were different, we found that *h* = 50 nm would be suitable because of the high quality of SP wave and a reasonable FWHM equal to 3.449 deg.

Figure 5 shows the influence of grating width (*w*) as it increased from 15 to 70 nm on the performance of the proposed sensor. As shown in Figure 5a,c, the resonance angle became smaller with increasing width, moving from 83.07 (*w* = 15 nm) to 71.22 (*w* = 70 nm) deg. In addition, FWHM and MRR exhibit similar changes as the value of *w* changed, as shown in Figure 5b. With grating width (*w*) increasing from 15 to 50 nm, the SPR curves possessed narrower FWHM and smaller MRR. However, after further increasing the width, the MRR increased slightly. 

In Figure 6, it can be seen that the resonance angle, FWHM, and MRR were strongly dependent on the grating period (*Λ*). As shown in Figure 6a,c, the resonance angle redshifted from 71.68 (*Λ* = 60 nm) to 77.79 (*Λ* = 100 nm) deg. In addition, the SPR curves became broader with the value of *Λ* increasing from 60 to 140 nm. The MRR slowly decreased during the 60–100 nm range and quickly increased when the value of *Λ* increased further. The feasible value of *Λ* = 100 nm was obtained through the trade-off between FWHM and MRR to ensure strong excitation of SPs and a reasonable value of FWHM.

### 3.4. Performance Analysis and Comparison

**Figure 7:**
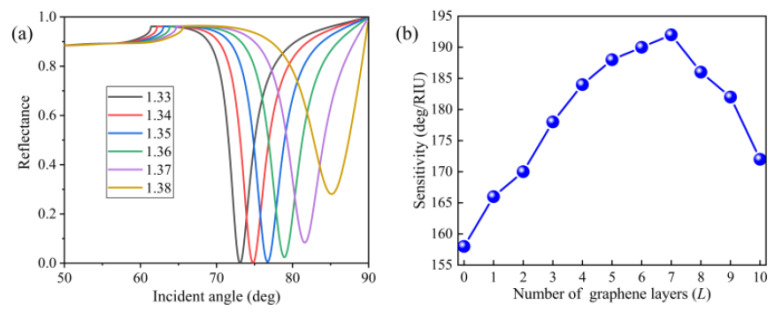
(**a**) Variation of reflectance with respect to the incident angle of the proposed sensor with monolayer graphene. The refractive index of sensing medium changed from 1.33 to 1.38. (**b**) Variation of sensitivity with respect to different number of graphene layers (*L*) when the refractive index of sensing medium was 1.33 + Δn.

Based on the above analysis, we examined how the structure’s geometrical parameters affected the performance of the sensor. Taking into consideration smaller MRR and a feasible value of FWHM, the final optimal structural parameters were determined as follows: upper and lower silver film thickness *T* = 15 nm and *m* = 10 nm, respectively; grating height *h* = 50 nm; grating width *w* = 50 nm; and grating period *Λ* = 100 nm. The SPR curves of Ag-only, Ag grating–Ag, and Ag grating–Ag–graphene configurations are presented in Figure S1 in the Supplementary Materials. The SPR sensors consisting of Ag gratings possessed smaller MRR, larger FWHM, and higher sensitivity when the refractive index of analyte changed from 1.33 to 1.335, as shown in Table S1. Note that the Ag gratings play an important role in this device because the coupling effect between graphene and plasmonic gratings can be significantly enhanced under optimized conditions. According to Table S1, the Ag grating–Ag and Ag grating–Ag–graphene structures showed sensitivity enhancements of 38.6% and 45.6% compared to the Ag-only SPR sensor. In order to illustrate the excellent performance of the proposed sensor, Figure 7 shows the variation of the reflectance with respect to the incident angle and the influence of the number of graphene layers on sensitivity. From Figure 7a, we can see that the resonance angle moved toward a larger incident angle with increasing refractive index of the sensing medium. The resonance angles were 73.10, 74.8, 76.72, 78.93, 81.61, and 85.14 for $n_{s}=1.33$, $n_{s}=1.34$, $n_{s}=1.35$, $n_{s}=1.36$, $n_{s}=1.37$, and $n_{s}=1.38$, respectively. It is obvious that there was a dramatic change in the resonance angle when the refractive index of the sensing medium changed from 1.33 to 1.38, which demonstrates that the proposed sensor is sensitive to a slight change in refractive index of the sensing medium. From Figure 7b, it can be seen that the sensitivity was strongly dependent on the number of graphene layers when the refractive index of the sensing medium was 1.33+Δn. Figure 7b also indicates that the sensitivity continuously enhanced with the number of graphene layers increasing from 0 to 7, and the maximum sensitivity (192 deg/RIU) was obtained at *L* = 7. The sensitivity then began to decrease when *L* became larger than 7. It should be mentioned that the incident angle ranged from 0 to 90 deg. With the number of graphene layers increasing, the reflectance curve shifted to a larger angle, but its limit was 90 deg. We could obtain the highest sensitivity due to the largest change of resonance angle when the layers of graphene increased to the optimized value. However, when the layers of graphene were larger than the optimized value, the change of resonance angle decreased, which resulted in a gradual decrease in sensitivity.

**Figure 8:**
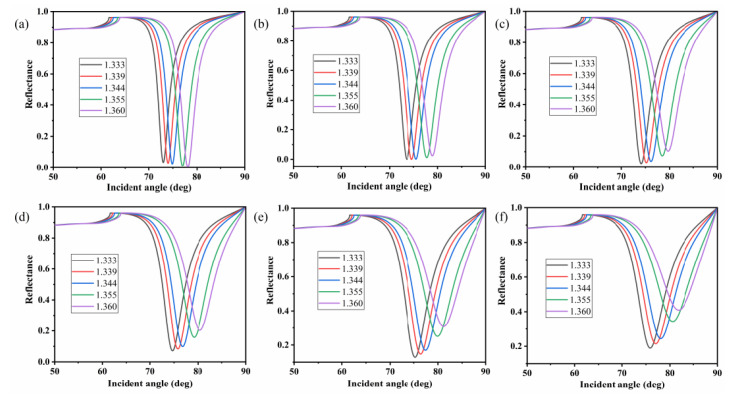
Reflectance curves of the proposed sensor corresponding to different graphene layers: (**a**) without graphene; (**b**) monolayer; (**c**) bilayer; (**d**) three layers; (**e**) four layers; (**f**) five layers. The refractive index of the ethylene solutions changed from 1.333 to 1.360.

In this study, ethylene glycol solutions as the sensing medium were obtained by mixing ethylene glycol and distilled water with different volume ratios to test the sensor ability [50]. The proposed sensor, illuminated with light at a wavelength of 633 nm, was used to measure ethylene glycol solutions at various volume ratios with a refractive index range of 1.333, 1.339, 1.344, 1.355, and 1.360. Figure 8 presents the reflectance curves corresponding to different graphene layers. It is obvious that there were distinct SPR adsorption dips. In contrast, the reflectance curves with Ag-only layer is shown in Figure S2 in the Supplementary Materials. From Figure S2, it is obvious that the resonance angle changed from 67.7 to 71 deg with the refractive index range of 1.333, 1.339, 1.344, 1.355, and 1.360. Figure 8a shows the reflectance curves without graphene. It can be seen that the resonance angle moved to larger incident angles with increasing refractive index of the sensing medium, and the resonance angle redshifted 5.08 deg in the range of 1.333 to 1.360. In contrast to Figure S2, Figure 8b–f exhibits evident resonance angle shift with increments of analyte refractive index. The resonance angle shifted 5.34, 5.57, 5.75, 5.91, and 5.94 deg for the proposed sensor with different graphene layers from monolayer to five layers, respectively. Moreover, the SPR curves clearly broadened from monolayer to five graphene layers, which can be characterized by the FWHM.

**Figure 9:**
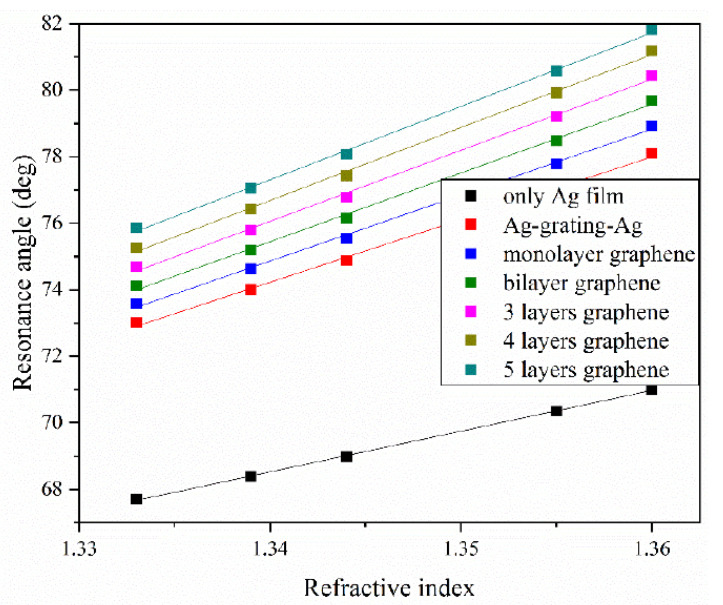
Linear fitting curves of resonance angle as a function of refractive index with different configurations. The refractive index of the ethylene solutions changed from 1.333 to 1.360.

In order to describe the sensing performance more intuitively, the resonance angles are plotted as a function of the sensing medium refractive index for Ag-only and various graphene layers in Figure 9. The squares represent the measured resonance angles for different refractive indices, and the lines indicate the linear fit. The sensing sensitivities for zero to five graphene layers were 188.444, 198.192, 206.624, 213.553, 219.242, and 220.666 deg/RIU, respectively. The sensing sensitivity for the Ag-only configuration was 122.446 deg/RIU. It is obvious that the sensitivity increased gradually due to the existence of gratings and graphene. In the case of five graphene layers, the sensitivity was enhanced to 220.67 deg/RIU, which is 17.10% and 80.21% more than for zero graphene layer and Ag-only, respectively. In addition, the linearly dependent coefficients *R*^2^ corresponding to the cases of zero to five graphene layers were 0.99798, 0.99785, 0.99772, 0.99769, 0.99792, and 0.99843, respectively, which adequately demonstrate that there was a good linear relationship between the resonance angle shifts and refractive index changes. Therefore, the refractive index of the sensing medium can be calculated through the fitting equations if the solution concentration is unknown. To sum up, Table 1 shows a quantitative comparison of the recent literature with the results obtained in this work. As can be seen, the SPR sensor based on graphene and Ag gratings proposed in this paper showed comprehensive performance with the highest sensitivity.

## 4. Conclusions

To conclude, a novel SPR sensor incorporating graphene and subwavelength Ag gratings is proposed in this paper. The effects of metal film thickness and grating structural parameters, including grating height, grating width, and grating period, on the sensing performance, such as FWHM, MRR, and resonance angle, were thoroughly studied. The results indicated that near-zero MRR ($2.9\times 10^{-6}$) and a narrow FWHM (about 3.5 deg) could be obtained by optimizing the geometrical parameters. Under configuration with optimized geometrical parameters, the effect of the number of graphene layers on sensitivity was investigated. The maximum sensitivity of the proposed sensor could reach 192 deg/RIU, which is a significant enhancement compared to the Ag-only SPR sensor. In addition, ethylene glycol solutions with different refractive indices were detected. The results showed that the sensitivity of the sensor reached 220.67 deg/RIU, and the proposed sensor had excellent linearity between the resonance angle and refractive index. Moreover, the proposed device can be readily fabricated using nanolithography techniques (for instance, electron-beam lithography followed by graphene transfer) and then experimentally characterized. The proposed sensor shows great potential for extensive biochemical applications with high performance.

## Figures and Tables

**Table 1:** Comparison of different sensing designs.

Design	Sensitivity (deg/RIU)	FWHM (deg)	MRR	References
Au–Ag bimetallic film	54.84	--	0.1	[17]
Au–Si–graphene	134.6	17.975	--	[33]
Rh–Ag–graphene	220	10.204	--	[34]
SnSe–graphene	94.29	7.454	0.0032	[35]
Graphene tri sheets	121.67	3.30	--	[36]
Au–graphene	46	5.52	--	[47]
Graphene–MoS_2_–Al	190.36	--	--	[48]
Ag–graphene	91.76	1.754	--	[49]
Graphene–Ag grating	220.67	7.093	0.19	This work

## Supplementary Materials

The following are available online at https://www.mdpi.com/2079-4991/10/11/2125/s1, Equations (S1)–(S7): The calculation of the reflectance; Figure S1: The reflectance curves of Ag-only layer, Ag grating–Ag, and Ag grating–Ag–graphene configuration when the refractive index of analyte was 1.33 (G represents graphene); Table S1: MRR, FWHM, and sensitivity of sensors with different structures; Figure S2: Reflectance curves of the proposed sensor corresponding to Ag-only layer. The refractive index of the ethylene solutions changed from 1.333 to 1.360; Figure S3: Linear fitting curves of resonance angle with refractive index for Ag-only configuration. The refractive index of the ethylene solutions changed from 1.333 to 1.360.
