# Supplementary material for: Sensitivity-Enhanced SPR Sensor Based on Graphene and Subwavelength Silver Gratings
Source: Nanomaterials (Basel). 2020 Oct 26;10(11):2125. doi: 10.3390/nano10112125 (PMC7693912; doi:10.3390/nano10112125)
Supplement: Supplementary file 1 [file nanomaterials-10-02125-s001.pdf]

Supporting Information

Sensitivity-Enhanced SPR Sensor Based on Graphene and Subwavelength Silver Gratings

Lu Kong [1,2], Jiangtao Lv [1,2,*], Qiongchan Gu [3], Yu Ying [4], Xiaoxiao Jiang [3,*] and Guangyuan Si [1,2,*]

[1] College of Control Engineering, Northeastern University at Qinhuangdao, Qinhuangdao 066004, China; kl13043130659@163.com
[2] College of Information Science and Engineering, Northeastern University, Shenyang 110004, China
[3] Hebei Key Laboratory of Micro-Nano Precision Optical Sensing and Measurement Technology, Qinhuangdao 066004, China; guqiongchan@163.com
[4] College of Information & Control Engineering, Shenyang Jianzhu University, Shenyang 110168, China; yingyu@sjzu.edu.cn
* Correspondence: lvjiangtao@neuq.edu.cn (J.L.); jxx316@mail.ustc.edu.cn (X.J.); siguang@mail.ustc.edu.cn (G.S.)

To obtain reflectance, we employed the transfer matrix method and Fresnel equation to perform detailed analysis. In the calculations, the first boundary of the tangential field was assumed as $Z_1 = 0$, and the relationship between the tangential field of the last boundary Z_{N-1} and the first boundary Z_1 is given as follows:

$$\begin{bmatrix} U_1 \\ V_1 \end{bmatrix} = M \begin{bmatrix} U_{N-1} \\ V_{N-1} \end{bmatrix} \quad \text{(S1)}$$

where U_1 and U_{N-1} represent the tangential components of the first and the last layers in the electric fields, respectively, while V_1 and V_{N-1} denote the corresponding components in magnetic fields. *M* refers to the characteristic matrix of the *N*-layer model. For the *p*-polarized light, the characteristic matrix is given as follows:

$$M = \prod_{k=2}^{N-1} M_k = \begin{bmatrix} M_{11} & M_{12} \\ M_{21} & M_{22} \end{bmatrix} \quad \text{(S2)}$$

with

$$M_k = \begin{bmatrix} \cos\beta_k & (-i\sin\beta_k)/q_k \\ -iq_k \sin\beta_k & \cos\beta_k \end{bmatrix} \quad \text{(S3)}$$

where

$$q_k = \frac{(\varepsilon_k - n_1^2 \sin^2\theta_1)^{1/2}}{\varepsilon_k} \quad \text{(S4)}$$

$$\beta_k = \frac{2\pi n_k \cos\theta_k (Z_k - Z_{k-1})}{\lambda} = \frac{2\pi d_k}{\lambda}(\varepsilon_k - n_1^2 \sin^2\theta_1)^{1/2} \quad \text{(S5)}$$

Thus, the four elements M_{11}, M_{12}, M_{21}, and M_{22} of the matrix *M* can be calculated. According to the Fresnel equation, the complex reflection coefficient r_p of *p*-polarized incident electromagnetic field can be described as follows:

$$r_p = \frac{(M_{11} + M_{12}q_N)q_1 - (M_{21} + M_{22}q_N)}{(M_{11} + M_{12}q_N)q_1 + (M_{21} + M_{22}q_N)} \quad \text{(S6)}$$

where q_1 and q_N represent the relative components of the first layer and the N-th layer, respectively. Therefore, the system reflectance R_p for the p-polarized incident light can be obtained as follows:

$$R_p = |r_p|^2 \tag{S7}$$

The SPR curves of Ag-only layer, Ag grating–Ag and Ag grating–Ag–graphene configuration are presented in Figure S1. The SPR sensors consisting of silver gratings possessed smaller MRR, larger FWHM, and higher sensitivity when the refractive index of analyte changed from 1.33 to 1.335, as shown in Table S1. Note that the silver gratings play an important role in this device because the coupling effect between graphene and plasmonic gratings can be significantly enhanced under optimized conditions. According to Table S1, the Ag grating–Ag and Ag grating–Ag–graphene structure showed sensitivity enhancements of 38.6% and 45.6% compared to the Ag-only layer SPR sensor, respectively. The reflectance curves with Ag-only layer is shown in Figure S2. It can be seen that the resonance angle changed from 67.7 to 71 deg with the refractive index range of 1.333, 1.339, 1.344, 1.355, and 1.360. For comparison, Figure S3 plots the linear fitting curve of resonance angle with refractive index for Ag-only configuration. The refractive index of the ethylene solutions changed from 1.333 to 1.360.

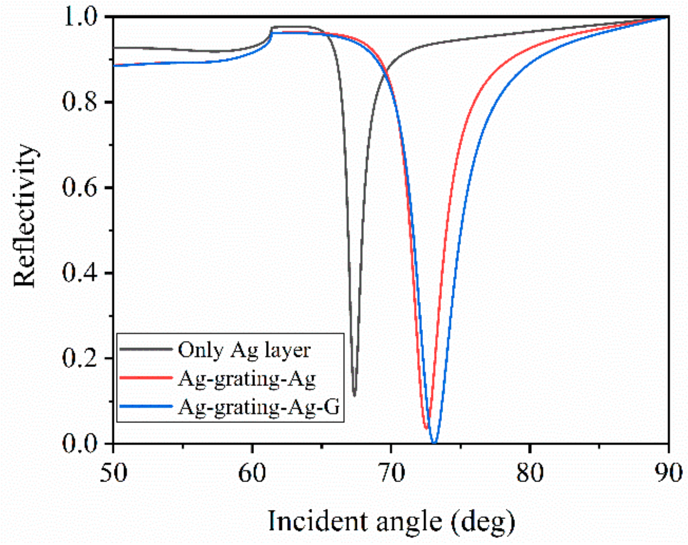

Figure S1. The reflectance curves of Ag-only layer, Ag grating–Ag, and Ag grating–Ag–graphene configuration when the refractive index of analyte was 1.33 (G represents graphene).

Table S1. MRR, FWHM, and sensitivity of sensors with different structures.

Structure	MRR	FWHM (deg)	Sensitivity (deg/RIU)
Ag-only layer	0.1126	1.187	114
Ag grating–Ag	0.0364	2.722	158
Ag grating–Ag–graphene	2.929×10^{-6}	3.449	166

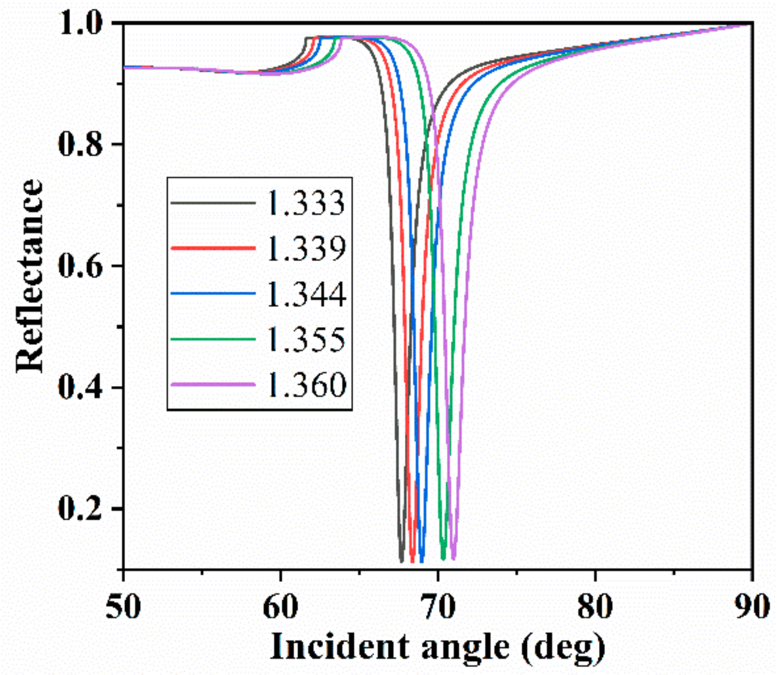

Figure S2. Reflectance curves of the proposed sensor corresponding to Ag-only layer. The refractive index of the ethylene solutions changed from 1.333 to 1.360.

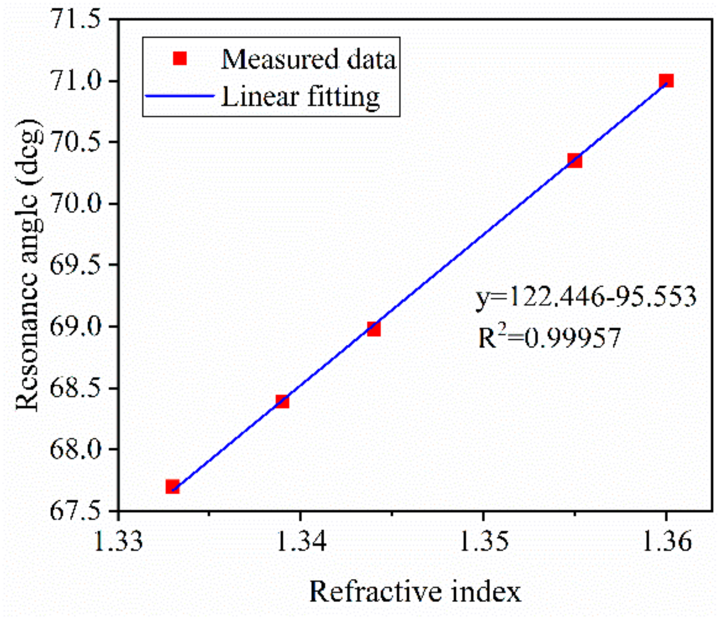

Figure S3. Linear fitting curves of resonance angle with refractive index for Ag-only configuration. The refractive index of the ethylene solutions changed from 1.333 to 1.360.
